# "The Hospital" Nursing Mirror

**Published:** 1899-01-21

**Authors:** 


"The Hospital" Nursing Mirror.

Being the Nursing Section of "The Hospital."

[Contributions for this Section of "The Hospital" should be addressed to the Editor, The Hospital, 28 & 29, Southampton Street, Strand, London, W.C., and should have the word "Nursing" plainly written in left-hand top corner of the envelope.]

### News from the Nursing World.

### ROYALTY AT ROMSEY.

According to promise, Princess Beatrice, accompanied by Miss Cochrane and Colonel Clarke, visited Romsey on the 11th inst. and opened the Royal Nursing Home that has been built as a memorial of the Queen's Diamond Jubilee. Unfortunately, the wet weather somewhat spoiled the effect of the pretty ceremony that had been arranged. The town was decorated, and in front of the Town Hall a platform had been erected which was draped with banners. Here the Princess, who had been met at the station by the Mayor, the Mayoress, and Mr. Basil Montgomery, was presented with an address. Her Royal Highness then proceeded to the pavilion adjoining the Nursing Home, where she accepted a bouquet, listened to a second address, and received a number of purses on behalf of the institution. After declaring the Home open the Princess unlocked the door with a golden key, with which she had been presented by Mrs. Montgomery, and inspected the new building. The Royal party then visited "Stanbridge Hall" and "Brooklands," the residences of Mr. Montgomery and the Mayor respectively, and returned to the Isle of Wight at five o'clock. The new Home, which occupies a freehold site in Cherville Street, has cost £575. The money has been raised by public subscriptions, Mr. and Mrs. Montgomery having exerted themselves to bring the undertaking to a successful issue.

### CHRISTMAS ENTERTAINMENTS.

With this number we end our descriptions of Christmas entertainments. The universal desire to make Christmas a season of rejoicing and festivity has never been more noticeable, and from all sides comes the tale of an abundance of dainties, a plethora of amusement, and a wide distribution of some one or other useful gift as a souvenir. The preparations for these celebrations of the festival of goodwill have entailed an enormous amount of labour, especially upon our nurses, who are often overworked to begin with. Intense interest has centred round our "sister," and our "ward"; the decorations and entertainments have occupied the thought of performers and onlookers months beforehand. Yet when all is happily accomplished and everyone is free to draw a long breath and talk it all over there is not much to say that is very new, or anything very striking that made "our entertainment" stand out with extraordinary interest to those who had not the pleasure of partaking of it. It is for this reason, therefore, that our pages have not been filled to overflowing with innumerable accounts of festivities, and not because we do not sympathise with those who have worked so hard and met with so great a success in arranging them. Many hearts have devised, many hands have worked out the means of expressing one divine principle, and in doing so they have carried peace and gladness into innumerable lives, and so in their labours they are themselves blessed and recompensed.

### FOR THE DARWEN NURSES.

Mr. C. P. Huntington, the Mayor of Darwen, being desirous of giving that town a memorial of his tenure of this office, has bought and furnished a house for a nurses' home. In order that his generous gift may be the nucleus of what may in time grow into a cottage hospital—an institution much needed in the district—he has invested the trustees with authority to sell the property and invest the proceeds in a larger house when circumstances warrant it. Mrs. Huntington is the president, and occupied the chair at the recent annual meeting of the association, when this gift was announced. She said that the financial position of the society was improving, and that people were awaking to the advantages it offered. Mrs. R. Eccles, the secretary, read the report, which showed that £401 had been spent, against the total income of £303 received. This statement seems to show that there is considerable room for improvement in the subscription list.

### THE DISTRICT NURSE.

The increasing recognition of the value of the district nurse could not be better illustrated than it was at a recent meeting of the Colchester Board of Guardians. The point under discussion was the sum to be granted to the Nursing Association, and the Rev. G. G. Brown mentioned, in advocating a liberal contribution, that he had a list of over 90 unions, each of which subscribed to the funds of the district nurses, the amount sometimes reaching £70 a year. The subscription voted to the Colchester District Nursing Association was £10. It is not generally known that the Local Government Board sanctions these grants when it is shown that paupers benefit by the ministration of the nurse. It is difficult to know who derives most advantage from the presence of a good nurse in the district—the chronic invalids, who, by her care, not only have their sufferings minimised but often are enabled thereby to remain in their home, and to retain their part in the family interests; the busy house-mother, who has neither time nor skill to do her duty by the invalid; the ratepayers and guardians whose pockets are considerably heavier for every patient kept off the rates; or, lastly, the nurse herself, who is able to earn an honourable livelihood in a noble calling.

### PRIZEGIVING IN EDINBURGH.

The opportunity of the nurses' annual festival at the Edinburgh Royal Infirmary was as usual taken advantage of to bestow the prizes awarded to those probationers who have distinguished themselves in the examinations. The event was looked forward to with much interest by the staff, and a number of visitors, amongst whom were the Lord Provost Mitchell Thomson, Mrs. Mitchell Thomson, Lady Struthers, and Miss Louisa Stevenson, were received by Miss Spencer, the lady superintendent, in Ward IV., which was arranged as a reception-room for the occasion. The Lord Provost distributed the prizes, and said that though "the nurses might not all obtain prizes in their profession, because there were very few, yet in doing their duty they had the consciousness they were performing a noble service which would not go unrewarded."

### THE BRAINTREE TROUBLE.

EVERY now and again there crops up the old trouble caused by differences of creed. This time it is the affairs at the Braintree Cottage Hospital that are in a tumult because the committee have dismissed a Roman Catholic nurse, "in consequence of the Roman Catholic convent being so near." Roman Catholics deal with circumstances like this more logically as a rule than Protestants, and, therefore, are to be commended. For instance, it is not probable that a Protestant nurse would be dismissed from a Romish institution on account of her faith, for the simple reason that it is unlikely she would have been appointed in the first place. In the event of such a suggestion, the authorities would rightly have said, "The majority of our subscribers and patients are Catholics, and their feelings ought to be considered in this matter; besides, the introduction of an officer of an antagonistic faith will open the door to disagreements, and the liability to such will increase in direct ratio to the fervour and conscientiousness of the individual." Both Catholics and Protestants alike would assent to the force of this argument, and thus there would have been no occasion for personal acrimony. If Roman Catholic nurses are objected to why appoint them? If they are not objected to what has the proximity of the convent to do with the question?

### THE STATUS OF TRAINING SCHOOLS.

THE question, "What constitutes a training school?" is often asked, but it is by no means easily answered. Many people think that the possession of a certain number of beds, the residence in the building of a medical officer, courses of instruction to the probationers by properly trained teachers would constitute this qualification. True, all these things are necessary, but one thing more is essential, and that is, recognition of any particular institution as a training school for nurses by the Local Government Board. Such is the lesson to be learnt from the state of affairs at Clonmel. Fifteen months or so ago the Guardians dispensed with the pauper nurses, and appointed in their stead a fully-trained nurse and a number of probationers. Dr. Crean, the medical superintendent, undertook to give lectures, and at the end of three years these young women were, upon passing a satisfactory examination, to receive certificates. The hopes of all concerned have been dashed to the ground by a letter recently received from the Local Government Board, which declares that "probationers cannot acquire the status of trained nurses by receiving instructions in Clonmel Infirmary." We cannot say whether the action of the Board is right or wrong in this case—that is for those before whom full evidence is laid. But it is only right that it should be pointed out that here is an infirmary connected with a fever hospital (the combined beds of the two numbering 215), with a trained resident nurse, and with a resident medical officer, the certificates of which are not recognised for the highest nursing posts by the central authority. Other institutions—and they are many—which propose solving the difficulty of obtaining nurses by training their own probationers should be warned that definite recognition of their status as training schools for superintendent nurses by the Local Government Board is the essential preliminary step. It is also only fair to impress upon the minds of young women intending to devote themselves to this work that they should make sure that the institution to which they give their services is thus recognised.

### SHORT ITEMS.

THE Crediton Sick Nurse Association has come out of a heavy year very well. The balance in hand is much the same as at the beginning of last year, in spite of temporary help having to be engaged on account of the illness of the district nurse.—A second nurse has been appointed to the Vale of Leven District Nursing Association. There is a balance in hand to meet the expenses thus incurred and to furnish the nurse's rooms.—It is reported with reference to founding a branch of the Colonial Nursing Association in Liverpool that Mr. Jones, of Messrs. Elder, Dempster, and Co., is endeavouring to arrange an influential meeting for the purpose, and that several eminent persons have been invited to attend.—Three public institutions share in the profits of the "Jubilee Nonsense Book." They are the Queen's Jubilee Nurses', the District Messenger Boys' Club, and the Prince of Wales's Fund. The share of the last named is £10.—A donation of £200 has been made by the Baroness de Hirsch Gerenth to the new nurses' home of the Chelsea Hospital for Women.—The Woolwich, Plumstead, and Charlton Nursing Association has received an anonymous gift of £5.—The Nursing Association of Barmouth, Dyffryn, and district is flourishing. The nurse has won golden opinions and the balance in hand is considerable.—The tragedy that has been hanging over the fate of Nurse Florence Bell is now solved. The body of the poor young woman has been recovered from the river near Hammersmith, and the evidence seems to show that death was caused by suicide. It will be remembered that Nurse Bell left the nursing home at Richmond to which she was attached to visit a patient in the vicinity, and has been missing for some time.—Two nurses belonging to St. Pancras Workhouse Infirmary, Dartmouth Park Hill, were severely burnt about the face and arms in consequence of a fancy dress worn by one of them taking fire. The accident happened at the New Year entertainment.—Nurses Flora Silver and Charlotte E. Hale, who were prepared at the Plymouth Workhouse Infirmary, have successfully passed the L.O.S. examination.—The sum of £19 7s. was collected for the Christmas Fund at the Barry District Nursing Association and Accident Hospital, £9 4s. 6d. of this being spent on good things for the season, and £10 2s. 5d. paid into the Special Nourishment Fund for 1899.

### Hints on the Home Nursing of Sick Children.

By J. D. E. Mortimer, M.B., F.R.C.S., formerly Surgical Registrar, &c., at the Hospital for Sick Children, Great Ormond Street.

(*Continued from page* 157.)

### OBSERVATION OF THE PATIENT.

Much may be learnt by careful observation of the patient. A small child will often give a vague or misleading reply when asked "where it hurts"; perhaps, because it has heard people complain of headache, when ill will do the same, no matter where the pain really is. Older children will sometimes exaggerate, or even in rare instances wilfully deceive, or may conceal their troubles from fright or dislike of medicine or from shyness or modesty; and one meets now and then, even in quite little children, curious "hysterical" simulation of real disease. Constant watching is essential when infants or young children are suffering from acute disorder, as they often change very rapidly. The following are the points which in case of a child should be specially noticed: The demeanour (apathetic, excited, or otherwise unnatural). The posture or attitude assumed when left free to move, and in which there seems to be most comfort — equally so any which seems to have the reverse effect; any movements of the head or limbs, especially if repeated. Any apparent loss of power, stiffness, or signs of tenderness. The expression of the face and colour of the lips; frowning, working of the nostrils, twitching or drawing of the lips; much dilatation, contraction, or inequality of the pupils; squint. The tongue and mouth, especially as to dryness, fur, presence of thrush, swelling of gums. Any discharge from the nose or ears; any swelling of the glands of the neck; any disinclination to swallow, or apparent pain on doing so. The skin, as to colour, heat, moisture, and presence of any eruption. The hands and feet, whether cold or warm, and whether the fingers and toes are in a natural position. The rate and regularity of the pulse may be sometimes better taken by feeling the temporal artery (just in front of the ear or along the margin of the hair) than the radial at the wrist.

The temperature should be taken in the fold of the groin or in the rectum; this is more reliable than the axilla, and a time should be chosen before the child is disturbed, as for washing or changing. It is dangerous to put a clinical thermometer in a child's mouth. In the young the temperature may suddenly run up for some very trifling cause, and if there are no other bad symptoms and it does not remain high there need be little alarm. The temperature of a small, delicate child may also be raised by too much external heat (hot water bottles). The respiration, whether easy, laboured, catchy, or accompanied by unusual signs such as snoring, crowing, sinking in above the collar-bones on inspiration, &c. The rate should be taken by watching the rise and fall of the clothes, or gently placing the hand on the abdomen or chest. Cough: the frequency, loudness, degree of looseness, and other characters. Young children seldom expectorate.

Crying: it must be clearly understood that babies do *not*

"Only do it to annoy,
Because they know it teases,"

and that there are a great many reasons besides hunger, although a certain class of mothers seem to have a fixed idea to the contrary. There is no cry without significance, and the greatest care should be taken to find out the cause in order that it may, as far as possible, be remedied, and this may in the same child be quite different from one time to another. For example, the infant may have been overfed, or may be thirsty, tired, cold, sleepy, or uncomfortable from tightness or wetness of clothing or from lying in a cramped position. Pins and fleas are frequent sources of irritation. The cry of real hunger is more or less emphatic according to the strength, and is accompanied by suggestive movements such as sucking the fingers or efforts to sit up. That arising from other discomforts is more fretful, whilst in a fit of temper (not often seen under six months) the cry becomes a "roar," and the baby holds its breath and alternately kicks out and stiffens itself. If there has been fright or "nightmare" there may be clutching at the nurse, and the gaze is directed to some fixed point or wanders about as is in search of something which has disappeared. In case of griping the cry is almost passionate; there are tears shed and often there is vomiting or discharge of wind, the baby draws up its legs and writhes, stops, and then goes on again. When the pain is in the chest there is every few minutes a short stifled cry, generally without tears. In extensive inflammation of the lungs and severe rickets, the child cannot spare breath to cry at all. Teething causes a state of peevishness with occasional outbursts; earache or other sharp pain a series of screams, the hand being perhaps put to the affected part, and the pain evidently made worse by anything which disturbs it, such as swallowing or sudden raising of the head in case of the ear, or movement in case of a joint. I may also mention the characteristic sudden scream of meningitis, and the hoarseness due to laryngitis. The cry of exhaustion is a pitiful whine, and may be conjoined with such signs as a subnormal temperature, or sinking in of the fontanelle or membranous part of the skull. Having made sure that none of the foregoing causes, or any similar ones exist, there remains a very common one, and that is the cry of habit, to which I have already referred.

The condition of the abdomen, soft or hard, distended or hollow. The evacuations from the bowels, as regards frequency and nature, their colour and consistence, and any abnormality, such as the presence of particles or lumps of milk curd, blood, mucus, or worms. A blackish colour may be due to the medicine. At least one good specimen should be kept for the doctor to see. Any vomited matter should be similarly dealt with, and the nurse should notice whether the sickness comes on soon after taking food or seems to occur independently.

Sleep: whether sound or broken by dreams, startings, difficulty in breathing, &c., whether the child awakens naturally or remains for a time in a dazed or agitated condition.

The urine: note unusual colour, smell, or deposit and pain (if any) accompanying the act. A baby should always be "held out" at regular intervals. A specimen may be obtained from a very young or unconscious patient either by the catheter or by putting plain absorbent wool or a cup inside the napkin.

(*To be continued.*)

### The Pension Fund Nurses.

### MISS BURNS' WEDDING GIFT.

Since going to press last week the following nurses have sent subscriptions towards the congratulatory address to be presented to Miss Burns upon her marriage by the members of the Pension Fund: E. A. Grant, H. Platt, Ethel Duncan, No. 560, E. C. Vernet, C. A. Hatton, V. E. Wood, M. McCartney, M. A. Wylie, S. A. Swift, T. E. Manning.

### Training in the Provinces.

(*Continued from page* 159.)

### ROYAL HANTS COUNTY HOSPITAL (108 Beds).

### Terms of Training.

Candidates for training at the Royal Hants County Hospital, Winchester, should be between 22 and 36 years of age. They are required to enter at first on a trial of three months, during which probationary period they have to provide their own uniform. If accepted at the end of this time the probationer signs an agreement for three years, one year of training and two in the service of the hospital. A premium of ten guineas is required from probationers, who receive no payment during their first year, salary beginning at £12 the second year and increasing to £18 the third year, with an extra allowance if during the last year they are employed in private nursing. If a nurse wishes to withdraw from her engagement before the expiration of the three years she must give three months' notice and forfeit £5. After gaining their certificate and completing three years' service nurses on the staff receive a yearly salary of £24. Sisters receive £30 per annum. Laundry and indoor uniform are provided by the hospital for all the staff.

A certain number of paying probationers are also received for one year's training on payment of thirty guineas, or for shorter periods by special arrangement. Four guineas are paid on entrance, and the balance at the expiration of the first month.

Certificates are given at the satisfactory completion of the three years' engagement. Lectures are delivered to the nursing staff by members of the medical staff and by the matron. A complete term of three years' training is an essential qualification for promotion to the position of ward sister, ordinary probationers being eligible for such promotion, if otherwise competent, after three years' work in the hospital. Special probationers are only eligible if they have had two years' satisfactory training elsewhere and one year in the hospital.

Night duty is taken for periods of not longer than three months at a time, in alternation with day duty.

### Hours On and Off Duty.

Day nurses are on duty on week-days from 7 a.m. to 8.30 p.m. Two and a half hours daily are allowed to nurses for recreation, and three hours to sisters, in addition to which half an hour is allowed in the morning for lunch and dressing, half an hour for dinner, and twenty minutes for tea. Off duty times for probationers are from 11.15 a.m. to 1.15 p.m., or from 2 30 to 4.30 (including tea); for nurses from 2.30 to 5 p.m. (including tea), or from 6 to 8.30 p.m. On Sundays probationers go on duty ten minutes later in the morning, and nurses half an hour later. Sisters are on duty from 8 a.m. to 8.30 p.m., with three hours off during that time, on week days from 5.30 to 8.30 or from 3 to 6 p.m., and on Sundays from 10 to 1 or from 6 to 9 p.m. These hours are in addition to meal times. Days off are at present given as and when it is found convenient, but it is hoped that very shortly this leave of absence may be more definitely fixed. The committee endeavour to arrange for leave of absence aggregating six weeks in each year, for each nurse, not more than four weeks holiday being taken at one time.

Night nurses' hours on duty are from 8.20 p.m. to 7 a.m.; on Sundays, 7.30. Their recreation hours are from 8.15 to 11 a.m.

### Meals.

All meals for the day nurses are served in the dining-room. Sisters have their lunch, tea, and supper sent to their own sitting-rooms. Allowances of butter and sugar are given out once or twice weekly to each nurse, but these stores are kept in a special and suitable place near the dining-room. The hours for meals are as follows: Nurses and probationers breakfast at 6.40 a.m., on Sundays at 7.10; lunch is between 9.30 and 10.30 a.m., dinner at 1.15 and 2.30 p.m., tea at 4.15 for nurses on duty, and supper at 8.30 p.m. Sisters breakfast at 7.45 a.m.; their dinner is at 1.15 p.m., and supper at 8.30 p.m. Night nurses have supper at 7.50 p.m., and breakfast at 7.45 a.m., with lunch at 11, before they retire to bed.

### SWANSEA GENERAL HOSPITAL, SWANSEA (130 Beds).

### Terms of Training.

Candidates for training at the Swansea General Hospital should be between 21 and 35 years of age. If accepted after a month or two months' trial they enter the hospital for a term of three years, one of training, and two further years in the service of the hospital, a certificate being granted at the satisfactory completion of the engagement. No premium is required from ordinary probationers, and salary begins the first year at £10, rising to £12 the second year and to £14 the third year. Staff nurses are paid £18 per annum, and sisters £30 to £35. There is a private nursing staff attached to the hospital, on which nurses may be appointed after three years' training and experience, receiving a salary of £25 per annum. Laundry and indoor uniform are provided for the nursing staff, and outdoor uniform also in the case of the private nurses.

A limited number of paying probationers are received for not less than three years' training, paying a fee of £12.

Lectures are given by members of the medical staff to the nurses, and examinations are held. Night duty is taken for not longer than three months at a time, and for not more than six months during one year.

### Hours On and Off Duty.

Day nurses and probationers are on duty in the wards between the hours of 7.15 a.m. and 8.30 p.m. From these hours must be deducted time for meals and recreation. Probationers are off duty on alternate days from 2 to 4 p.m. and from 5 to 6 p.m. Staff nurses are off duty twice a week from 5. 30 to 10 p.m., one afternoon each week from 2 to 10 p.m., and on every alternate Sunday from 4.30 to 10 p.m. On the other Sunday they do not come on duty till 12.45 p.m. Sisters go on duty in the morning at 8.15, and leave their wards at 9 in the evening. Their times off duty are the same as the staff nurses'. Annual leave is, for probationers, 14 days, for sisters one month.

Night nurses are on duty from 9 p.m. to 8 a.m. In winter they take their recreation from after their dinner at 8.30 a.m., till 12.15 p.m.; in summer, from 6 to 8.15 p.m.

Probationers have leave every alternate Sunday afternoon from 4.30 to 9 p.m., and one whole day once a month till 9.30 p.m. They are occasionally required to go on duty till 9 a.m. on these days off.

### Meals.

All meals, except the midnight meal for those on night duty, which is served in the ward kitchens, are laid in the dining-room. Lunch is served in the dining-room from 9 to 9.30. No "allowances" are given, unless to the sisters, for tea, which they have in their own rooms. The night nurses have an allowance sent to the ward kitchen for midnight meals. The hours of meals are as follows: Day nurses and probationers' breakfast is at 6.45, luncheon is from 9 to 9.15, dinner at 1.15, tea at 4 and 4.30, supper at 8.30. The sisters breakfast at 7.45, their dinner is at 12.30 p.m., tea at 4, supper at 8 p.m. Night nurses have their breakfast at 8.30 p.m.; they have "substantial refreshment" at 2.30 a.m., and dinner at 8.30 a.m.

### A Book and its Story.

### TONY DRUM.

REALISM has to bear the burden of much that is repulsive to many minds, and even when our realistic writers have genius to soften the crudities of which they write and the artistic touch which compels our admiration of even the most unsavoury subjects, it is almost invariable that the result is morbid, depressing, or repulsive. It is, however, fortunate that realism can at times, in the hands of a true artist, be only absorbing and pathetic, and those two expressions alone describe the story of "Tony Drum,"* by Edwin Pugh. Edwin Pugh writes with a full knowledge of his subject, and it is to his keen sympathy and accurate observation that we owe the most pathetic child-hero of recent fiction. The story is sad, but that the story of a crippled little East-end waif with a happy-go-lucky, tipsy father and a mother of much the same character could be otherwise is not to be expected. Yet it is with no sense of depression that we close the book, but with gratitude to the hand that wrote it. Humour and pathos lie side by side in its pages, and intermingle without any suggestion of incongruity.

Tony Drum was born in Garden Row, a street on the left bank of the Thames. The people who live in Garden Row are mostly—to quote the author's word—"connected," more or less closely, with river business. They work, live, and die hard. "Tony's parents were poor. His mother had been a domestic servant; his father was a musician and philosopher. But philosophy having no marketable value, Michael Drum was forced to use his talent for music. He blew a flute in the gusty streets for a meagre livelihood. . . . He was none of your showy rascals. He was a man, middle-aged, commonplace, of spare figure, and faltering gait. . . . In his youth he had been picturesque enough. . . . During his romantic years he had tended sheep on the hill slopes for an inexorable father. His life was ill-suited to his ardent nature, and he grew to hate it. He longed to taste London," and to London Michael went, leaving his more placid existence of the sheep hills behind him. At last we find him again—a husband and a ratepayer in two rooms in Garden Row, and it is here where we are introduced to the hero of Mr. Pugh's drama of low life, at the epoch in his life when "Tony was a tiny, deformed miracle of five. He had a great shock head, obliquely set between high, pointed shoulders, a thick, humped body, and rickety legs." . . . "It was known throughout the Row that Tony was foredoomed to early death—that was the one happy fact of his existence." Tony had a sister, his senior by a year or so. The affection which the two small creatures of adversity bore to one another is the happy theme throughout the story. The two were "rarely apart; he never appeared without her." Tony was the pet of the Garden Row Mission Hall. "He was very devout, and would listen to the longest homilies with the rapt face of an angel. Once he asked his sister, 'God lives in Heaven, don't He?' 'He does when He's at home,' Honor said, striving to cope with the great demands of the subject. 'But generally He's out and about doing good.' 'Oh!' gasped Tony. 'He's everywhere, like, you see,' said Honor." Tony often asked questions, quaint outcomes of the child's wondering mind. "Don't ask so many questions," said his mother on another occasion, and Michael Drum added, "We must send the boy to school."

Thus his education was begun. Life was easy and slow in the infant school of St. Anselm's. Learning was administered in wee sips, and the discipline was lax. Often on a summer afternoon, when the grey floor was a mosaic of sunshine, half the tiny scholars would be asleep, and the teacher herself in a state of great drowsiness, so that her voice dwindled to a low drone, very soothing to the dulled senses of the children. Tony's schooling came to a sudden end after one of his periodical bouts of sickness, and the boy knew not if he was glad or sorry; but when he was well enough he "crept downstairs every morning and sat upon the doorstep in the sun. He became a spectator of life. He saw much that puzzled him, but of one thing he was certain, the world was very kind." A crisis in Tony's life arrives in the chapters headed "He Plays at Kiss in the Ring" and "He Catches Glimpses of the Feminine Heart." In the latter chapter Tony finds himself the happy recipient of a little girl's affection. He has a sweetheart whom he devotedly adores. But Miss Carrie Green, the golden-haired object of his adoration, only raises the boy to the seventh heaven of ecstacy to as suddenly desert him. The estrangement, like the courting, was without many preliminaries. It happened in the following manner. "'Its like this,' she began, 'You see, lots of gals wouldn't have you at all! . . . They'd say, "I wouldn't never have Tony Drum for a bloke. You must be hard up!" they'd say.' . . . 'Then I renounce you!' he said. 'Oh, don't, Tony.' 'Yes,' he said, fiercely; 'I give you up. Good-bye for ever, Carrie Green.'" This little drama was followed by a reconciliation, but Miss Carrie Green eventually bestows her affections elsewhere. This was not the last of the boy's heart-burnings. His sister went away to live with the distant grandfather, to become a "lady." The children correspond with each other, and their letters, too long for quotation, are exactly the letters one would expect them to write. But letters do not solace Tony. He became very ill. Some kind good friends of the child's ask him what would be his greatest treat, and he should have it, and he answers, to look at the sea.

"In life Tony was very intimate with Death, and when his time came Death called to him with the voice of a friend. He never saw the sea. Saturday was the day fixed for his departure from London; on Friday he died. Excitement had made him very ill, and all day he lay in a fevered stupour, watching the cloud armies of the sky. . . . 'Good-night, Father,' he said, turning on his side. 'I shall be awake very early in the morning.' And he fell asleep."

* "Tony Drum." By Edwin Pugh. (London: Heinemann. 1898.)

### Appointments.

ROYAL BERKSHIRE HOSPITAL, READING.—Miss Mary R. Easton was appointed Matron and Superintendent of the nursing department of this hospital on the 10th inst. She was trained at the Nightingale School, and was afterwards sister at St. Thomas's Hospital. She has been matron of the Hospital for Children and Women, Waterloo Bridge Road, S.E., since October, 1896.

GOVERNMENT HOSPITAL, BAHAMAS.—Miss Elizabeth Jackson has been appointed Matron of this hospital. She was trained for three years at the New Infirmary, Birmingham. She has since held posts in the Eastern Hospital, Homerton, and in the Mogden Hospital, Isleworth. Miss Jackson holds the L.O.S. certificate.

GWELO HOSPITAL, RHODESIA.—Miss Mary E. Blackett has been appointed Matron of this hospital. She was trained at the London Hospital, and was subsequently night sister and afterwards sister of the women and children's ward at the Poplar Hospital for Accidents.

### Everybody's Opinion.

[Correspondence on all subjects is invited, but we cannot in any way be responsible for the opinions expressed by our correspondents. No communication can be entertained if the name and address of the correspondent is not given, as a guarantee of good faith but not necessarily for publication, or unless one side of the paper only is written on.]

### A LETTER OF THANKS.

Mrs. A. Ward, Indian Industrial School at Battleford, Sask., N.W.T., Canada, writes: I beg to thank you for so very kindly inserting my appeal for a copy of The Hospital. It was responded to most generously. I was both glad and sorry. I was glad to find what good, kind, thoughtful hearts there are. I felt sorry that so many came, as I felt as if I was taking what might have been of such help and value to others. I hope, too, that your valuable paper will be blessed by a largely increased circulation, thus carrying its usefulness far and wide, as well as at home. May I ask if you would kindly convey my thanks to those who so kindly sent me their copies of The Hospital. I am deeply grateful to all. I may add, any literature is welcome in this lonely, lonely place. It cheers, comforts, and instructs. We are 100 miles from the nearest railway station. Freighters, &c., fetch and carry. Things are dear and inferior. Five Indians died on Sunday night (December 18th) on one reserve close by.

### THE HEALTH OF NICE.

"Traveller" writes: The travelling world is at present agitated by the statements for and against the sanitary state of Nice, and the public is bewildered by reading the most positive announcements as to the presence to an alarming degree of typhoid fever in that favoured town, only to be nonplussed the next day by an equally positive denial. I have read all that has been said by persons in authority and also made private inquiries, and I cannot find that there is any real cause for anxiety. There have been isolated cases, of course, as there always are in all large towns, only we do not hear of them, and when it is considered how the infection of typhoid is carried care and precaution will minimise all danger. Never drink water unless it is boiled, and see to the boiling yourself; be equally strict about the milk; boil it yourself always; indeed this is a thing that should always be done on the Continent. It is a little trouble, perhaps, but you then feel safe and free from anxiety. In all my travelling I never allow myself to be too tired or too lazy to boil my drinking water and all milk. It is a mistake to drink soda water and kindred fluids, as they, too, are usually made on or near the spot.

### NURSING IN SOUTH-WEST AFRICA.

E. Billington writes: I am writing to you as I think you may like to know something of my nursing experiences in the Congo. During my first term in Congo I had a midwifery case (twins) soon after my arrival, and afterwards helped to nurse several cases of malarial fever; all these in the circuit of the Baptist Missionary Society, Bwemba Station, Tchoumbiri, Haut Congo, Congo Independent State, and principally (excepting the midwifery case) at Stanley Pool. Dr. A. Sims, who has just gone to England on furlough, was a near neighbour, so I generally could refer to him for any help. My first furlough was a long one of nearly three years on account of my own health, which was a good deal upset by my first husband's death on the voyage home, and then by the health of my little boy. As soon as I felt justified in leaving my little boy I returned to the Congo with Mr. Billington, reaching the coast in January, 1893, and our station in March of the same year. I generally do what medical or surgical work there is to be done amongst the natives after our morning service, having a sort of miniature dispensary; and frequently I have a few patients in the villages near us who are not able to come to me. Lately I have had a few cases of pneumonia, which I am glad to say are recovering, though chest troubles are often quickly fatal with these people. During the last two months the number of those coming to me for medicine, dressings, &c., has varied from four to eighteen. A few have chronic ulcers, some dysentery, some worms; once I was asked to take a slug from a native gun out of a man's hand; I have it yet, a bit of ironstone from a hill at the back of our station! One patient came to me with a worm crawling about in his eye. It came out on the point of a darning needle, and measured 1¾ inches. I bottled the worm and showed it to Dr. Sims next time we went to Stanley Pool, and he assured me that such worms were quite common, and rather difficult to get out. Sometimes there are what seem to be syphilitic cases, and with some of these I have to get my husband to help me. I think our worst trouble among the natives is the sleeping-sickness, and we have not yet found any cure for it. Sometimes a tonic seems to put it off for a little while. Just now we have two cases on the station; they come to us sometimes to make sure of ending their days in peace, because if they stayed with their friends or owners they would be thrown in the river as soon as they got too feeble to care for themselves. Sometimes we get a passing missionary to nurse with malarial fever, and occasionally a State officer. In March last we were called to the next mission station, thirty miles up-river, to help with a fever which had already been several days about 105 deg. I think it was a puerperal fever, and it lasted five days longer, then ending fatally, with a temperature of over 107 deg. We have no hospital here, and no doctor near. Of course, I do not profess to be up to date as nurses at home can be. All the nursing I did at home was done with the one idea of gaining experience to be used out here, and I have been very glad of it all. I see The Hospital regularly, and often get help from it. I have given you these particulars so that you may have something like a correct idea of my work.

### Minor Appointments.

Whitchurch Cottage Hospital, Shropshire.—Miss Florence Davis was appointed Head Nurse here on the 11th inst. She was trained at the Guest Hospital, Dudley, where she has remained ever since, excepting for a temporary absence in 1896 (during alterations at the Guest Hospital), when she took temporary superintendent duty at Wolverhampton Hospital, having charge of the fever block. She has been in charge of the children's ward, of the large female ward, of the gynæcological ward, and of the operating theatre, and was, during the last part of her time there, senior sister.

Liverpool Stanley Hospital.—On December 16th Miss Janet Cooke was appointed Night Superintendent of this hospital. She was trained at the Guest Hospital, Dudley, and at the General Hospital, Nottingham. Miss Cooke has been sister and theatre sister at the Royal Infirmary, Sheffield, and sister in charge of the patients in a private ophthalmic home.

Infectious Diseases Hospital, Foleshill, near Coventry.—On January 4th Miss Sarah Armson was appointed Nurse Matron at this hospital. She was trained at the Coventry and Warwickshire Hospital, Coventry, and at the City Hospital, Birmingham; she has also held the post of charge nurse at the Hartlepool Hospital, Hartlepool.

Chorlton-cum-Hardy, Manchester.—Miss M. M. Wight was appointed Queen's Nurse of the above district on December 1st. She was trained at the Liverpool Royal Infirmary, and for upwards of two years had charge of a district in connection with her training school.

South Stoneham Union Workhouse.—Miss E. Mason, who was trained at the General Hospital, Nottingham, has been appointed working Superintendent Nurse of the above infirmary. She has been employed in the Portsea Island Union Infirmary and in the Epsom Workhouse.

Plumstead Infirmary.—On the 5th inst. Miss M. A. Foster, who was trained at St. George's Infirmary, Fulham Road, was elected Head Nurse of this infirmary. Miss Foster has held posts in St. George's Infirmary and at Camberwell Infirmary.

Newry Union Infirmary.—On January 14th Miss Eleanor Gordon, who was trained at the Belfast Union Workhouse, was appointed Superintendent Nurse of the above.

### Royal British Nurses' Association.

As a decided contrast to what has happened on some previous occasions, the meeting of the British Nurses at the Medical Society's Rooms on Friday evening, 13th inst., was peaceful almost to dulness. There were but 28 present all told, and the only matter about which there was not absolute and voiceless unanimity was the question of the suspension of one of the members against whom, it was alleged, convictions for perjury and theft had been recorded. Very naturally, and quite properly, the Council was reluctant to take so serious a step as the removal of the accused one's name from the roll of membership without the clearest proof that she had erred from the path of righteousness; while at the same time convinced that the adoption of that course was their plain duty, however painful it might be, if the facts were as alleged. There was therefore a considerable amount of quiet but earnest discussion before the final decision to refer the matter back to the Executive Committee to report to the next meeting of the Council was come to. The chair was taken by Dr. Bezly Thorne.

Mr. Langton, the hon. treasurer, presented his report for the past quarter, and stated that after the settlement of all liabilities there remained in hand a bank balance of over £70, in addition to the £189 for investment—a considerable improvement upon their former position. A very satisfactory feature was the position of the *Nurses' Journal*, which was now more than paying its way, an announcement received with hearty applause. Altogether this was the most satisfactory statement ever presented to the Council of the Association, and he felt that their thanks were due to those who had assisted them in their financial difficulties.

Mr. E. A. Fardon, the medical hon. secretary, presented the report of the Executive Committee. During the past three months 40 nurses had been registered, 40 members had been elected, three had died, and 32 had withdrawn. The names of 145 had, in accordance with the usual custom, been removed from the roll for neglecting to pay their subscriptions for three years. The committee announced with regret two vacancies on the General Council and one on the Executive Committee. Miss Hephzibah Walker's health prevented her retaining her seats on the council and the committee, while Miss Robertson, who was elected at the last meeting, was unable to attend on account of the distance from London at which she resides. Miss G. A. Leigh had resigned the post of acting secretary, which she had held since November, 1897, and had accepted the post of secretary to the Corporation. This announcement was greeted with applause. A profit of £22, exclusive of £8 12s. 6d. contributed to the Convalescent Fund had been realized on the first year of the monthly issue of the *Nurses' Journal*. The accounts for the Convalescent and Benevolent Funds showed that, in addition to £79 set aside for investment, the sum of £50 had been received for the benefit of members in distress, of which £12 had been expended in grants, leaving £38 in hand. It was proposed now to amalgamate these funds. The holder of one of the Helena pensions, Mrs. Mary Ann Sandford, died in October last. The Privy Council having decided that the names of nurses could not be published under the title of a "register," it had been determined to print the roll of members in a more official and complete form, with the names of those who were not members separately. The first sessional lecture, which was well attended, was given by Miss Rosalind Pritchard on December 9th on "Our Hospitals." The committee announce the following arrangements for the remainder of the session: In January, Dr. Bezly Thorne will lecture on "The Nurse's Part in the Prevention of Tuberculosis"; in February, Mrs. Garrett Anderson on "Vaccination"; and in March, Mrs. Clare Goslett on "Sanitary Science for the Nurse's Profession." The annual conversazione, usually held in December, has this year been unavoidably postponed, but the committee hope to announce the date at which it will be held shortly.

In proposing the adoption of the report, Mr. Fardon said it really seemed as if their downward course had been arrested; the horizon was distinctly brighter, and they had every reason to hope that the present year would see a great improvement in their position and prospects generally. It was extremely gratifying that their *Journal* had been placed upon a paying basis, for there was no doubt an organ of that kind was most desirable.

The adoption of the report having been seconded and carried unanimously,

The Hon. Medical Secretary proposed the adoption of the new regulations, observing that they involved no new departure in policy, the changes made being simply matters of convenience. The regulations are as follows:—

The General Council.

1. The meetings of the general council shall be held on the fourth Friday in January, April, and October. The date of the meeting following the annual general meeting shall be fixed by the Executive Committee immediately preceding the annual meeting.

The ordinary meetings shall be held at 5 p.m., unless otherwise ordered by the Executive Committee.

2. At any ordinary meeting fifteen shall be the quorum, excepting when a larger number is necessary under the charter.

3. Notice of any special or ordinary meeting shall be sent out at least five days before the date of meeting; the said notice to contain the agenda.

4. No new regulation for the conduct of the general council meetings shall be proposed, nor any old regulation altered or rescinded, unless notice of such proposed addition or alteration shall have been given fourteen days previously, in writing, to the secretary, and shall have been duly noted on the agenda paper.

5. The order of business at the meetings of the general council shall be as follows:—

(*a*) To read and confirm the minutes of the previous meeting.
(*b*) To receive and consider reports from the Executive Committee.
(*c*) To consider any motion of which notice shall have been given in writing, fourteen days previously, to the secretary.
(*d*) To do such other business as may be necessary.
(*e*) To receive any notice of motion to be brought before the next meeting.

The Executive Committee.

1. The ordinary meetings of the Executive Committee shall be held on the third Friday in each month, excepting August and September.

The ordinary meetings shall be held at five p.m., unless otherwise ordered by the committee.

Note.—When Good Friday falls on the third Friday in the month, the date of meeting shall be fixed by the committee at their previous meeting.

2. At any ordinary meeting of the Executive Committee five shall be the necessary quorum.

3. No new regulation of the Corporation shall be proposed at any meeting of the committee, nor any old regulation altered or rescinded, unless notice of such proposed addition or alteration shall have been given at least ten days previously, in writing, to the secretary, and shall have been duly noted on the agenda paper sent to each member of the Executive Committee.

4. The order of business at the meetings of the Executive Committee shall be as follows:—

(*a*) To read and confirm the minutes of the previous meeting.
(*b*) To receive and consider reports, if any, from chairman, treasurer, hon. secretaries, and secretary. To receive and consider reports from the Registration

Board, Editorial, Finance, and other sub-committees.

(c) To discuss any subject under consideration at, and adjourned from, any previous meeting.

(d) To consider any motion of which notice has been given.

(e) To do any other business.

(f) To receive any notice of motion to be brought before the next meeting.

THE BENEVOLENT FUND.

1. All existing funds for granting charitable assistance to members shall be amalgamated in one, to be called "The Helena Benevolent Fund," and from this fund any annuity shall be paid.

2. The Helena Benevolent Fund shall, subject to the control of the Executive Committee, be administered by the Finance Committee, which shall hold a quarterly meeting on the Tuesday next before the meeting of the Executive Committee, preceding the meeting of the General Council.

3. A meeting of the Finance Committee may be summoned at any time to consider applications for assistance.

4. Cases of urgent distress may be relieved at the discretion of the nurse honorary secretary, acting together with the honorary treasurer, provided that the amount so granted to any one person does not exceed the sum of £2.

5. Members of the Association of not less than two years' standing shall be eligible to receive a grant:—

(a) If temporarily disabled by sickness from following their calling.

(b) If in need of rest, or change of air, after illness.

(c) If in temporary distress from any cause except want of employment.

6. The committee shall have power to make continuous grants to members under the name of Helena Annuities, provided always that the funds at the disposition of the committee, or the subscriptions promised, suffice to meet such grants for a period of two years in advance from the time when they are first granted.

7. Members of not less than five years' standing shall be eligible to receive Helena Annuities if totally incapacitated from work by age or infirmity.

8. The Helena annuitants shall be nominated by the Finance Committee and elected by the Executive Committee.

These having been adopted without discussion, the council proceeded to consider a motion by Miss Scott, suggesting that the Association should be represented in provincial towns by lady consuls, who should themselves be members of the Association, with the hope that individual nurses might be benefited and the growth of the Association increased. In the unavoidable absence of Miss Scott, the resolution was proposed by Mr. FARDON, who said he felt sure that it would be of advantage to all if all over the country there were representatives of the Association able to give advice and assistance to nurses visiting or residing in the locality. These ladies would occupy in relation to nurses the position of our consuls abroad with regard to British subjects generally. The idea had met with unanimous approval. It was not altogether a new one, since they had always had power to arrange for local centres. These had in practice, however, been found to be cumbrous and unworkable. They wanted in each place some one energetic person with tact and knowledge to make the idea a success, and she would then really become the nucleus of a local centre.

Dr. ALDERSON seconded the proposal, which he considered a most useful one and calculated to promote *esprit de corps.*

The CHAIRMAN also spoke in warm approval of the proposition, and said it was one which would have been carried out long ago, but that the energies of the Association had been diverted into other channels—a remark that brought smiles to the faces of those who recalled some former meetings—and it was unanimously decided to empower the Executive Committee to take the matter up and carry it out.

The next proposition was a painful one, namely, to consider the advisability of suspending one of the members. his lady, the HON. MEDICAL SECRETARY said, was elected a life member in 1887, and in 1890 she was granted the badge. Since then various allegations with regard to her character had been made, and steps were taken to find out the ground for these, but without success. Then on two separate occasions—in September last and again later still—paragraphs had appeared in the public press stating that since she had become a member of the Association she had been guilty of theft. Registered letters were sent to the address she had given drawing her attention to these accusations, and asking what steps she was taking with regard to them. These letters, however, had been returned, and they had been unable to find her whereabouts. Since then a letter had been received—not from a private source, but one which he was not allowed to make public—stating that in 1881 the lady in question had been sentenced to nine months' imprisonment for perjury, and that at the Lewes Assizes in April, 1884, she was sent to prison, with hard labour, for fifteen months for theft.

Considerable discussion followed, in which most of those present took part. Some members felt that it was imperative that so undesirable a person should no longer remain a member of the Association; others felt that an anonymous letter—for such it was as far as they were concerned—and a paragraph in the *Nursing Record*, the paper in which the statements appeared, were not sufficient justification for so serious a step. Mr. FARDON offered to show the letter to any member privately, and at length, on the suggestion of the CHAIRMAN, a sub-committee, consisting of Miss Entwisle, Miss Merritt, Miss De Pledge, Dr. Outterson Wood, and Dr. Francis Hawkins, was appointed to examine the evidence—the letter read by Mr. Fardon in fact. Eventually it was proposed by the Hon. MEDICAL SECRETARY (Mr. Fardon), and seconded by the VICE-CHAIRMAN (Miss Thorold), that the name be erased from the roll, but an amendment proposed by Miss DE PLEDGE, and seconded by Miss ENTWISLE, as a result of having read the letter referred to, that the name be suspended from the roll of members pending the verification of the evidence which had been seen by the sub-committee, was carried by 14 votes to 7. On its being pointed out that 14 votes were not sufficient even for suspension, the amendment was made the substantive motion, proposed by Miss THOROLD, seconded by Miss ENTWISLE, and carried by 21 votes. The matter was then referred to the Executive Committee to report to next meeting of the council, and after the proposal to approve a list of institutions had been withdrawn for further consideration the meeting closed with the customary votes of thanks.

### Presentations.

MISS WILLIAMS, who has been the district nurse at Exmouth for some years, and who has now accepted an appointment in Edinburgh, has been the recipient of two handsome gifts from the friends amongst whom she has been working. The residents of the parish of Littleham-cum-Exmouth have given her a purse containing £30 and an illuminated address, and those of Withycombe a purse of £15 and an illuminated address.

MISS ETHEL M. HOYLE was presented by the medical staff of the Ulster Hospital, Mount Pottinger, Belfast, with a handsome silver bowl and an address on the occasion of her resigning the office of Lady Superintendent. The sisters and nursing staff gave her at the same time an afternoon tea-kettle. Miss Hoyle had been lady superintendent of the hospital for the last four years.

### OUR CONVALESCENT FUND.

ANOTHER kind gift of 2s. to our Convalescent Fund! It is with much pleasure that we thank Miss H. M. Walker for it.

### Travel Notes.

By Our Travelling Correspondent.

Rules in regard to Correspondence for this Section.—All questioners must use a pseudonym for publication, but the communication must also bear the writer's own name and address as well, which will be regarded as confidential. All such communications to be addressed "Travel Editor, 'Nursing Mirror,' 28, Southampton Street, Strand." No charge will be made for inserting and answering questions in the inquiry column, and all will be answered in rotation as space permits. If an answer by letter is required, a stamped and addressed envelope must be enclosed, together with 2s. 6d., which fee will be devoted to the objects of the "Hospital Convalescent Fund." Any inquiries reaching the office after Monday cannot be answered in "The Mirror" of the current week.

### VII.—A SHORT HOLIDAY IN THE ARDENNES.

I have been asked to give some account of foreign places suitable for nurses to spend their holidays in, and I shall do so with much pleasure from time to time. It is all-important, especially if engaged in private work, that a nurse should pass her leisure times in scenes as absolutely new and different as possible. It is not only advantageous to herself, but immensely so to her patients, who will often be amused with accounts of what befel her in strange and unexpected circumstances. It is a serious drawback in private nursing that the attendant's conversation and "outlook" is so limited. She goes from house to house, and from one sick bed to another on her errands of mercy without having had time or opportunity to refresh her mind and body with new scenes, new faces, new sounds even, and new experiences. It stands, therefore to reason and common sense that she cannot be so healthful and invigorating a companion as is desirable. Let me beg of you all to think seriously of this view of the matter, and try to arrange to take your holiday in some foreign place and with a congenial companion. You would not, of course, go alone, and having a friend halves many expenses, such as carriages, &c.

I wrote a series of articles a year or two ago demonstrating how it was possible to spend a week on the Continent, in France, Belgium, Holland, or Germany for £5 with ease and comfort, and if you can be absent a fortnight or three weeks the cost is much less in proportion, because the initial expense of the journey is the same for a long or short period. This week we will take Dinant on the Meuse as our spot to consider. Easter or Whitsuntide are ideal seasons for the green and sunny country of the Ardennes, and the roads are perfect for cycling. If you belong to the C.T.C. you can take your bicycle into Belgium free, if not you must pay duty. If you do not cycle or do not care to take your machines, walking is delightful, and very long rounds can be made by help of the railway lines recently constructed. If you have a fortnight clear, exclusive of travelling days, you may reckon your expenses at £7 inclusive. Take your ticket second-class return, £1 16s. 7d., which will be *via* Antwerp and Brussels, to Namur. Leave the train there in favour of the boat, so that you may enjoy the lovely scenery of the Meuse between Namur and Dinant. The little steamer lands you under the grey old overhanging houses which are most picturesquely reflected in the water, and which are backed by the fine church with its singular spire, and the heights crowned by the citadel behind. There are many cheap hotels I can tell you of if you decide to go, but if means permit of it the Tête d'Or stands first in my affections. It is a nice, homely, little hotel, with a glass vestibule in front, where enthusiastic cyclists congregate and compare notes as to their respective achievements, each individual lending but scant attention to his neighbour's records.

Dinant makes the best headquarters for exploring the Ardennes if time is limited. It is not now so primitive as when I knew it first, 15 years ago; increased railway facilities have brought many tourists and hordes of cyclists, but what it has lost in simplicity it has gained in solid comfort. To those interested in sketching or photography it presents endless attractions. It was my good fortune to be staying there with some eminent artists, and, as is their kindly way, they were most helpful in pointing out charming views and picturesque nooks around. One of your first excursions must be to the château of Veve-celles. I was first moved to visit Dinant by reading Mrs. Macquoid's very fascinating book, "Through the Ardennes." I should advise you to get it from the library before you go. Her account of Veve-celles is the best I have met with anywhere. Its history is as mysterious, romantic, awe-inspiring, and weird as that of Glamis. A grim silence has set its seal upon the past, and though horror and mystery seems breathing everywhere in its deserted rooms, no one knows any definite fact connected with the cause of its decayed and desolate condition. It lies about six miles from Dinant, and I believe you can go now by train as far as Wanlin, thereby cutting off a mile or two. A carriage all the way for two persons costs about 12 francs.

A very delightful and extended walk of four or five hours may be taken thus: Start from Dinant by the picturesque hamlet of Anseremme. At Pont a Lesse cross the river by primitive ferry (you pull yourself across by a chain) and visit the magnificent and frowning Château of Walzin, which, crowning a perpendicular rock some 150 feet in height, throws its reflections upon the deep and sullen waters below. Pass on to the village of Furfooz and return by the Château of Veve-celles; the whole walk through wooded and mountainous paths is entrancing. N.B.—It will not do for cyclists. The first time I took that walk we had in our party a very old gentleman, who intrepidly announced his determination not to be left behind. He held out well till within two miles of home, when two of our young men, to his wrath and indignation, seized him with a strong hand and carried him on their backs by turns. It was far too long a walk for any but the young and robust, but the valiant old man was none the worse in the end. He wisely went to bed on reaching the hotel, and was supported by the application of some mysterious toothsome cordial by our excellent host, Monsieur Disierè. The next day we persuaded him to repose on the sofa, wrapped in a flowered dressing-gown which might have adorned the noble person of Sir Roger de Coverley, and we ladies hovered around offering the incense of sundry cups of tea and delicate flattery on his pedestrian prowess.

A much shorter but very pretty walk is up the valley behind the citadel to the Fonds de Leffe, a romantic, rocky ravine, full of quaint water mills. The abbey is no longer the resort of holy men, but is turned to the base uses of a glass factory.

A delightful excursion is the Grottoes of Han, 20 miles distant. The whole journey can be made by rail, but the scenery by road is so fine that bicyclists will thoroughly enjoy it. It must be noted, however, that very often it is impossible to visit the grotto in the spring on account of the swollen state of the streams. Inquire, therefore, first as to the possibility of entrance. The ruins of Montaigle, some of the finest in Belgium, are not far from Dinant, also the village of Bouvignes, sleeping under the crumbling tower of Crêve Cœur, where once dwelt fierce rivals of the Dinantais. This last is now a squalid little place largely given up to the overbearing occupation of the domestic porker who stalks majestically in and out of the ancient hovels with an air of conscious proprietorship. In the little time-worn church, mass is still said once yearly for the souls of three beautiful ladies of Crêve Cœur, who when the castle was besieged by

Henry II. threw themselves from the tower rather than fall into the hands of the conquerors. Further on come the ruins of Poilvache, still frowning and formidable even in decay. There are many other places to visit such as La Roche, Rochefort, &c., but my space is limited, and I must not linger longer to-day. I think you will learn to love the little town as I do, it is so quaint and the people so simple and friendly, whilst the church holds one's affections tenaciously. It is so peaceful to wander in at gloaming time and see the dim mysterious aisles lighted only by a taper here and there and peasants kneeling about waiting their turn at the confessionals.

HINTS FOR TRAVELLERS.

Immense comfort will be derived from the following old maidish arrangements on a long night's journey. Have several bags of different sizes, made envelope shape, of the commonest art muslin, at 2d. a yard, let them be of different colours, and into them put your slippers, wrappers, towels, boots, odds and ends, &c., to avoid the untidy appearance and distracting confusion of opening a hold-all, or bundle of wraps, and having all the contents rolling hither and thither.

Have some distinguishing mark, such as a scarlet crescent, or emerald green anchor, painted on all your luggage; it is an immense help, especially in diligence driving.

Take with you a folding drinking cup. They are made of aluminium, and fold up like a telescope to the dimensions of a full-sized pill-box. Cheaper kinds are made of indiarubber, but they taste unpleasantly.

Never travel in new shoes. Perhaps that seems a superfluous piece of advice, considering the imbecility of such a proceeding, but my eyes have beheld such imbecility.

Be sure that you have two francs worth of copper coins before starting.

### TRAVEL NOTES AND QUERIES.

NICE OR CANNES (Nemo).—I think, on the whole, Cannes is rather more expensive than Nice, but there is but little difference. Nice is larger, and therefore there is more competition and more choice of hotels. I think the Hotel Suisse would accept your terms, but they are rather low for the Riviera.

HOLLAND (Pimpernell).—This is not a favourable country for bicycling, on account of the pitched roads. The same may be said of North Belgium, but Southern Belgium and the Ardennes are charming countries for wheeling. Holland is a little expensive for travelling, but especially interesting to the artist and photographer.

INNSBRUCK (Omdurman).—Innsbruck is reached viâ the Hook of Holland, Cologne, and Munich. Cost, first class, £6 8s. 9d.; second class, £4 7s. 6d. There are several other routes, but this is the cheapest of all.

JERSEY (Dahlia).—It is no longer the ideally cheap place that it was; house rent has gone up considerably, and, taking one thing with another, I do not think that £300 a year would go further there than in England.

ROUEN (Spotted Dog).—Yes, there still remains much of artistic interest in Rouen, though the hand of the restorer has been heavy on it. The Cathedral, St. Ouen, and St. Maclov are among the finest specimens of French ecclesiastical architecture, and there are still many old streets left.

DRESDEN (Soap Bubble).—Dresden is far too cold in the winter for an invalid with delicacy of the lungs. This being the case, why not try Brussels?

THROUGH TO ST. PETERSBURG (Amethyst).—The quickest route is first-class £11 12s. 4d., second £8 3s. 2d. Only manageable for you in the summer.

VENICE (Byron).—Perfectly healthy if on the Riva degli Sciavoni, where you have the full benefit of the sea breezes. Hotel Danielli is excellent, but a little expensive. Try and make a pension arrangement by letter beforehand.

PISA (Tamerisk).—Kindly observe our rules and endeavour to limit your questions to matters of real importance. No, I do not think you would care for it in the height of summer. It is very much exposed to the sun and wind, and though full of artistic and architectural interest, I gather from your letter that these things are not in your line, and that you desire a gay watering place. Pisa is decidedly dull. Naples would suit you better, or even Spezzia, which is near to Pisa.

### Where to Go.

CHELSEA HOSPITAL FOR WOMEN, FULHAM ROAD, S.W.—The Duchess of Albany opens the new Nurses' Home on the 20th inst.

THE NATIONAL ORTHOPÆDIC HOSPITAL, GREAT PORTLAND STREET, W.—The Christmas tree for the patients and nurses will be lighted up at six o'clock on Saturday, 21st inst.

### Notes and Queries.

**The contents of the Editor's Letter-box have now reach d such unwieldy proportions that it has become necessary to establish a hard and fast rule regarding Answers to Correspondents. In future, all questions requiring replies will continue to be answered in this column without any fee. If an answer is required by letter, a fee of half-a-crown must be enclosed with the note containing the enquiry. We are always pleased to help our numerous correspondents to the fullest extent, and we can trust them to sympathise in the overwhelming amount of writing which makes the new rules a necessity.**

**Every communication must be accompanied by the writer's name and address, otherwise it will receive no attention.**

*Medical Dictionary.*

(164) Will you kindly tell me the names of one or two good medical dictionaries suitable for a nurse; also the price of each?—*N. E. L.*

The "Nurses' Dictionary of Medical Terms and Nursing Treatment," by Honnor Morten, 2s., from the Scientific Press, London. "Holbyn's Dictionary of Medical Terms" (10s. 6d.); publishers, Whittaker and Co., Paternoster Square.

*Massage.*

(165) Will you kindly inform me where and how I can obtain a knowledge of massage? I am a trained nurse, but have been nursing in fever hospitals for some years, and am now anxious to take up massage. 1. Can I give my services in return for lessons, or should I be able to have lessons while staying in London on a visit? 2. Which system would you advise me to follow?—*Inquirer.*

1. To take lessons whilst visiting London is the easiest plan. Consult our advertisement columns for addresses of reliable teachers. 2. Ask the medical men under whom you have nursed.

*A Registered Nurse.*

(166) "District Nurse" would like to know what advantages are to be gained by becoming "registered," and how to become a "registered nurse?—*C. M. C. B. C.*

Write to the Secretary, Royal British Nurses' Association, 17, Old Cavendish Street, W., for particulars.

*The Visiting Nurse.*

(167) Could a visiting nurse kindly give me some hints on how to begin as a visiting nurse in a city? I am fully trained (three years' certificate).—*A. M.*

Kindly tell me what steps a fully-trained and certificated nurse should take in order to become a visiting nurse?—*E. H.*

Settle in a neighbourhood where you are well known to the medical men, who will employ and recommend you. Ascertain what price per hour or visit your prospective patients can afford, and also what remuneration you must have in order to live and put by a little for the future. Reconcile the two, and have your terms printed on a form. Take care that each patient reads and accepts them before attending the case, and be careful that payments are made on the appointed dates.

*Ulcerated Leg.*

(168) Subscriber writes: Would you kindly tell me what would be the best dressing for an ulcerated leg for one who has always to be at work?

The best dressing for such a case must be decided by the surgeon in charge. A form of dressing which is very largely used at some of the hospital out patient departments is that by Unna's paste, but the mode of application and the care in ensuring asepsis, or at any rate cleanliness, are important factors in the treatment. Good success has also attended the use of formalin-gelatin, and careful support of the leg by bandaging. In both these methods the patients is encouraged to take exercise.

*"Toe Posts."*

(169) Can you oblige me with the address of the boot firm in London who make a toe-propped boot?—*Unfortunate Nurse.*

Do you not mean boots with toe posts? If so, the address is Messrs. Dowie and Marshall, 255, Strand, W.C.

*A Claim for Damages.*

(170) I am nursing a monthly case. Last Monday I was told that I could leave on Wednesday, provided my patient was well. Thereupon I made two appointments with patients, one in London on Wednesday and another in the South of England on Saturday, saying that unless they heard from me to the contrary by wire on Tuesday before eleven o'clock they might make their arrangements with their doctors, and I would accompany them. My patient was perfectly well herself, but at one o'clock she heard that the children's nurse would be unable to return from her holiday owing to having a poisoned foot. My patient immediately said she could not allow me to leave, as there was no one responsible enough to take charge of so young a baby. Asking my advice, I proposed that I should go to London for the day, and that she should pay my expenses, as she had been informed of my plans the day before, and had agreed to them. She declined doing this, although well able to afford it, but considered she had the first claim on my services, and felt much ill-used at my suggestion. I reminded her that the same patient had been put to great inconvenience through my being wired for eight days before my time, and that I was loth to put her off again. She only replied, "Let her get another nurse; she lives in a town, and I do not." However, I was unable to keep my appointment and have lost £3 3s. by it. Could I claim any damages for her not having kept her word? She could have obtained another nurse within an hour.—*Nurse Savilla.*

You should have kept your appointments at all costs (excepting actual danger to your first patient). You can certainly not claim damages, because you could have left if you had paid your own expenses up to town.

